# Spatial Accessibility Analysis of Snake Antivenom

**DOI:** 10.3389/ijph.2024.1606903

**Published:** 2025-01-03

**Authors:** Wenjie Hao, Lanfen He, Xingyue Song, Juntao Wang, Yanlan Hu, Yu Chen, Chuanzhu Lv, Shijiao Yan

**Affiliations:** ^1^ Hainan General Hospital, Haikou, China; ^2^ School of Public Health, Hainan Medical University, Haikou, Hainan Province, China; ^3^ Second Affiliated Hospital of Hainan Medical University, Haikou, China; ^4^ Sichuan Academy of Medical Sciences and Sichuan Provincial People’s Hospital, Chengdu, China

**Keywords:** antivenom, healthcare organizations, accessibility, snakebite, snakebite envenoming

## Abstract

**Objectives:**

To analyze the spatial accessibility of antivenom immunizing agents equipped hospitals in Hainan Province.

**Methods:**

This paper analyzes the spatial accessibility of medical institutions equipped with different types of snake antivenom using network analysis and two-step mobile search method, and evaluates the service level and spatial accessibility of medical institutions equipped with different types of antivenom immunizing agents in Hainan Province from the perspectives of both supply and demand.

**Results:**

The number of people in Hainan Province who need to spend more than 1 h to reach an *Agkistrodon Hyalys* antivenom, *Naja* antivenom, *Bungarus Multicnctus* antivenom, *Agkistrodon Acutus* antivenom equipped hospital, and equipped with antivenom for all species of snakes in the country is approximately 856,000, 231,300, 3,071,000, 2,666,000 and 4,721,000 people, respectively. In the results of accessibility of hospital beds/health technicians equipped with antivenom in Hainan Province, Haikou and Sanya cities had the highest accessibility.

**Conclusion:**

The accessibility of hospitals equipped with antivenom in Hainan Province is unevenly distributed, with areas of high accessibility in the southern and northern regions and accessibility in the rest of the country to be improved.

## Introduction

Snakebite is a widely distributed tropical and subtropical occupational disease that mainly affects agricultural workers in economically underdeveloped areas [1–3]. Snakebites kill between 81,000 and 138,000 people each year and leave up to 4 million others with long-term severe disabilities [4]. Untimely treatment of venomous snake bites can lead to serious complications, even amputation, disability and death, as well as psychological disorders such as post-traumatic stress disorder, seriously affecting the physical and mental health of the victim population [5]. In 2017 the World Health Organization (WHO) classified snakebite poisoning as a category A neglected tropical disease [6], and in 2019 the World Health Organization launched a global strategy for the prevention and control of snakebite envenomation in the hope of halving the number of deaths and disability cases caused by snakebite poisoning by 2030 [2]. This has led to research on appropriate prevention, implementable interventions and national and regional allocation of resources [7]. A central objective of the strategy is the need to ensure access to safe, effective and affordable treatment, such as antivenom and complementary medical services. Priority will be given to improving and strengthening the production, supply and distribution of life-saving antivenom and other commodities needed for the treatment of snakebite. Currently, the only effective treatment for venomous snake bites is passive immunotherapy with safe and effective antivenoms [8, 9].

The optimal time to administer antivenom is approximately 2 h after the bite [9], and the timing of access to formal treatment after a venomous bite and antivenom (ASVS) administration is critical in preventing patient deaths [10]. Early administration of antivenom is also useful in preventing complications such as acute kidney injury (AKI) [11], while there is an association between delayed consultation and increased severity of the condition, and early treatment with antivenom improves patient outcomes [12]. The World Health Organization has issued detailed guidelines on the production, quality control and regulation of antivenoms [13], long considered essential medicines. However, the availability of effective and safe antivenoms remains very poor in many parts of the world [14]. Achieving “universal access to safe, effective, quality and affordable essential medicines and vaccines” is included in United Nations Sustainable Development Goal (SDG) 3.8 and is a core component of Universal Health Coverage (UHC) [15]. Today geographic information systems (GIS) is a powerful technology for measuring geographic accessibility to healthcare [16]. An approach that is particularly well suited to modeling timely access to health services uses a least-cost pathway methodology that is based on local travel constraints (e.g., terrain, road networks, mobility barriers, modes of transportation, and speed). Analyze the distance cost and time cost from the point of need to the point of supply as a response to the accessibility of the point of supply. In line with the World Health Organization’s plan to improve the accessibility, affordability, efficacy, and safety of antivenoms, we use GIS to discuss the accessibility of antivenoms in Hainan Province. In 2016, the Central Committee of the Communist Party of China and the State Council issued the Outline of the “Healthy China 2030” Plan, stating that it is necessary to strengthen public health services covering the entire population as well as to provide high-quality and efficient medical services [17]. Against this background, the importance of conducting research related to medical services and public health services in China has increased significantly. An analysis of antivenom equipped hospitals from a spatial accessibility perspective will provide new information to the literature and will be important in improving the accessibility of antivenom. In this study: 1) A brief overview of antivenom availability in Hainan Province. 2) Spatial accessibility of antivenom equipped hospitals was calculated using network analysis and Gaussian two-step moving search method. This study describes the accessibility of antivenom-equipped hospitals in Hainan Province to provide a scientific basis for future allocation of antivenom resources.

## Methods

### Materials

#### Study Population and Location

Hainan Province is located in the southernmost part of China, with a total land area of 35,400 square kilometers, a sea area of about 2 million square kilometers [18], and a total population of more than 10,081,200 people in the province (mainly including Hainan Island and the Xisha, Zhong Sha, and Nansha Islands). Hainan Province currently has 19 cities and counties, including 4 prefecture-level cities, 5 county-level cities, 4 counties, 6 autonomous counties and 8 districts. Hainan Island has a tropical monsoon maritime climate with distinct wet and dry seasons. The rainy season generally occurs from May to October, and the dry season is from November to April. The province has 62.1% forest cover and is a natural habitat for many types of snakes. Especially in the central part of the city snake is the most, the central part of Hainan Province is surrounded by mountains, and is a big mountain, rainfall is sufficient, the mountain has a longitudinal forest of shrubs, suitable for the survival of snakes, such as Qiong Zhong, Wu Zhi Shan and Bao ting and so on.

#### Data

Vector data of Hainan Province’s administrative regions are from the standard map production with the review number: Qiong S (2023) 130 downloaded from the standard map service website of Hainan Survey and Mapping Geographic Information Bureau, with no modification of the base map, see Supplementary File 1. Haikou traffic network data from the National Center for Basic Geographic Information (NCBGI) 1:1,000,000 public version of the basic geographic information vector data. Multiple road files are combined and retained in a shapefile format.

The population distribution raster data for 2020 was obtained from the official World Pop website, and finally the World Pop 100 m*100m precision population raster data for 2020, corrected by the seventh census data at the street level, was used. World pop data is the number of people per 100 m^2^ scale grid estimated by the Random Forest method based on census data and multi-source auxiliary data by the Earth Data Research Institute of the University of Southampton, United Kingdom. The data can better reflect the distribution of the population, and therefore has been widely used in many fields such as urban planning and human geography [19]. In this study, in order to further improve the estimation accuracy of the population raster, the errors present in the original population raster data were corrected utilizing street-level 7th Census data. The specific correction method is: using the seventh census data in the number of street population divided by the number of the original grid summary population to get a linear correction factor, and then the original total population of each street will be multiplied by the correction coefficient, so that the total population of each grid in the street range word and the seventh census data are equal to the details, see Supplementary File 2.

A 2 km*2 km resolution grid was created for the study area with a search radius of 40km, and the generated center-of-mass grids were converted to vector data that were perfectly superimposed on the statistical grid for the study, where each grid had an area of 4 square kilometers. Centers of mass were generated for all these grids (squares), and then proximity tables were generated by neighborhood analysis to incorporate demographic and identifying information.

Snake antivenom are obtained by immunization of animals, and the antivenom in question is an immunoglobulin obtained by immunization of “horses”. The location and availability of health facility sites for antivenom were provided by Shanghai Salem Biological Company, and 25 antivenom availability sites were identified by geographic coordinate conversion using Gao De map. The number of beds and health technicians in medical units are obtained from the Health Statistics Yearbook of Hainan Province and the official websites of each hospital.

### Methods

This descriptive study was conducted using public health statistics and other information, and data were processed through the Geographic Information System (GIS) analysis tool (Geographic Information System or Geo-Information system, GIS) version 10.8.1. The GIS was used to create a fishing grid tool and the grid cells were set to 2 km*2 km, which ultimately gridded the study area into 8,866 population center of mass points (Since GIS software does not support tools for calculating the center of mass for raster data, it is necessary to convert the population raster data into point data to facilitate statistical analysis, so the point into which the raster data is converted is called the population center of mass point). Using the feature to point tool in Arc GIS, the surface file is transformed into a center-of-mass point vector file, and the population center-of-mass of the area is approximated by the cell center-of-mass to characterize the population center-of-mass of the area, and the number of people living in the cell is used as the demand in the accessibility calculation. The software is based on a least-cost model and calculates the commuting time between origin and destination using information about the transportation network, facility distribution points, and population center of mass points. Both the network analysis method and the two-step floating catchment area method were implemented by Arc GIS 10.8.1 software.

Information from different sources was used to analyze the geographic accessibility of the antivenom equipped hospitals. To meet the needs of the study, the sources and preparation of each data are described below. All data are custom projected according to the latitude and longitude of Hainan Province, the geographic coordinate system is GCS_WGS_1984; the datum is D_WGS_1984; the projected coordinates are Transverse Mercator, and the Central Meridian is 113.21266800; the prime meridian is Greenwich.

#### Network Analysis Method

OD cost matrix analysis is one of the network analysis methods, which is a method to simulate the representation of the real mesh structure using graph theory and operations research theory [20]. A complete network contains 4 basic elements: center, chain, node, and resistance. In the hospital accessibility study, the hospital coordinate point as the center, the road network from the population center of gravity point to the hospital as the chain, the road intersection as the node, and the time spent moving on the road as the resistance, in order to build a visual digital model for the simulation of the real path of the population center of gravity point to reach the hospital.

Based on the network analysis method, this paper uses the target starting point as the shortest distance/time from each population center of mass point, i.e., demand center, directly to the antivenom equipped hospitals to determine the ease of access from the population center of mass point to the hospitals and service utilization. This is done by obtaining the optimal path from the population center of mass point to each antivenom-equipped hospital and the optimal time cost by using the cost matrix between the population center of mass point and the antivenom-equipped hospital and the nearest facility point.

#### 2SFCA Method

The two-step floating catchment area method (2SFCA) is a method for evaluating accessibility. The method integrates the relationship between the supply of resources and the demand in the vicinity, and is simple and rigorous in its calculations.

In the first step, the first point j is used as the center of the hospital location, and the search radius is established with the limiting distance $d_{o}$ of the road network for people to go to the hospital as the search radius, and all the populations within the search domain j are summarized, and Gaussian functions are used to assign weights according to the law of distance attenuation, and these weighted populations are summed and aggregated to compute the ratio of supply to demand $R_{j}$ :

$$R_{j}=\frac{S_{j}}{\sum_{k\in \left(d_{jk}\leq d_{o}\right)}G\left(d_{ij}\right)D_{k}}$$ (1)

where $D_{k}$ is the population number of each population center of mass point k, d_jk is the road network distance between locations k and j. The road network distance from the demand unit to the nearest entrance is selected, and the unit k needs to fall within the search domain (i.e., $d_{jk}$ ≤ $d_{o}$ ); $S_{j}$ is the number of beds/health technicians in hospital j; $G\left(d_{ij}\right)$ is a Gaussian decay function considering the spatial friction problem, which can be expressed in the specific form:

$$G\left(d_{ij}\right)=\frac{e^{-\frac{1}{2}\times {\left(\frac{d_{o}}{d_{ij}}\right)}^{2}}-e^{-\frac{1}{2}}}{1-e^{-\frac{1}{2}}}\;\left(d_{ij}<d_{o}\right)$$ (2)

In the second step, with each population center-of-mass point i as the center, search for all the healthcare institutions j within the threshold range ( $d_{o}$ ), and summarize and superimpose the hospital location points to get the ratio of the number of beds/health technicians to the population of the hospitals in the final search domain ( $R_{j}$ ), i.e., we get the accessibility of the number of beds/health technicians in the i points ( $A_{i}$ ), and the larger the value means the higher the degree of accessibility: see Equation 3.

$$A_{i}=\sum_{\left.j\in \left(d_{ij}\leq d_{o}\right)\right)}{G\left(d_{ij}\right)R}_{j}$$ (3)

The supply/demand ratios for each supply radius were determined in turn from Equations 1–3, and finally the accessibility of the number of beds/health technicians for the study population was determined.

Threshold setting: Since antivenom provisioning requires cold chain and high requirements for hospitals and healthcare workers, the service radius was set to 40 km for analysis respectively.

Considering the differences in antivenom equipped hospitals and service capacity, hospitals were set to be reached by car, with a 15-minute drive time (40 km road network distance) as their spatial search threshold. According to the average peak hour travel speed of the road traffic operation index system in Hainan Province, the province’s roads are divided into the types of highways, expressways, arterial roads, feeder roads, other, internal, rural, bicycle, and pedestrian roads, and the travel speeds are set to 100, 80, 60, 20, 20, 20, 30, 15, and 5 km/h, respectively. The supply, demand and optimal path time results were substituted into the Gaussian two-step moving search method formula to calculate the accessibility of antivenom-equipped hospitals in Hainan Province and to analyze the number of beds available for 1,000 people and the number of health technicians for 1,000 people, respectively.

## Results

### Spatial Distribution of Medical Institutions Equipped With Antivenom in Hainan Province

A total of 25 hospitals in Hainan Province were equipped with snake antivenom, mainly in the northern and southern regions, of which 21 were equipped with *Agkistrodon Hyalys* antivenom, 14 with *Naja* antivenom, 11 with *Bungarus Multicnctus* antivenom, 11 with *Agkistrodon Acutus* antivenom and 5 with antivenom for all species of snakes. See Supplementary Table 1 for details.

### Results of Accessibility of Hospitals Equipped With Different Types of Antivenoms

Analyzing the accessibility of hospitals equipped with different types of snake antivenom based on the OD cost matrix in network analysis method.

#### Accessibility Results of 25 Snake Antivenom Equipped Hospitals in Hainan Province

As shown in Table 1, the number of population centers of mass to find the destination hospital was 8,707, and the number of population centers of mass that could reach the hospital in less than 30 min totaled 4,055, accounting for 46.6% of the total, covering an area of about 16,220 square kilometers, or about 3,703,700 people; The total number of population centers of mass greater than 30 min and less than or equal to 60 min or less is 4,106, or 47.2%, covering an area of about 16,424 square kilometers and about 4,077,100 people; The total number of population centers of mass greater than 60 min and less than or equal to 90 min or less is 498, or 5.7%, covering an area of about 1,992 square kilometers and about 587,300 people; The total number of population centers of mass greater than 90 min and less than or equal to 120 min or less is 48, or 0.6%, covering an area of about 192 square kilometers and about 38,000 people.

**TABLE 1:** Statistical results of optimal pathway accessibility of different snake antivenom (Haikou, China, 2024).

Type	Time(m)					Total
	≤30	30 < m ≤ 60	60 < m ≤ 90	90 < m ≤ 120	>120	
Equipped with						
Grid center point	4,055	4,106	498	48	0	8,707
proportion (%)	46.6	47.2	5.7	0.6	0	100
Area km^2^	16,220	16,424	1,992	192	0	34,828
population (thousands of people)	370.4	407.7	58.7	3.8	0	840.6
Equipped with *Agkistrodon Hyalys* antivenom						
Grid center point	3,855	4,159	645	49	0	8,708
proportion (%)	44.3	47.8	7.4	0.6	0	100
Area km^2^	15,420	16,636	2,580	196	0	34,832
population (thousands of people)	345.3	409.9	81.2	4.4	0	840.7
Equipped with *Naja* antivenom						
Grid center point	2,118	3,971	2,153	444	21	8,707
proportion (%)	24.3	45.6	24.7	5.1	0.24	100
Area km^2^	8,472	15,884	8,612	1,776	84	34,828
population (thousands of people)	231.8	377.6	209.0	21.6	0.7	840.6
Equipped with *Bungarus Multicnctus* antivenom						
Grid center point	1,799	3,683	2,510	654	61	8,707
proportion (%)	20.7	42.3	28.8	7.5	0.7	100
Area km^2^	7,196	14,732	10,040	2,616	244	34,828
population (thousands of people)	217.8	315.8	251.7	51.7	3.7	840.6
Equipped with *Agkistrodon Acutus* antivenom						
Grid center point	1,695	4,045	2,521	415	31	8,707
proportion (%)	19.5	46.5	29.0	4.8	0.4	100
Area km^2^	6,780	16,180	10,084	1,660	124	34,828
population (thousands of people)	224.1	349.9	245.0	18.1	3.5	840.6
Equipped with antivenom for all types of snakes in the country						
Grid center point	805	2,491	2,898	1794	719	8,707
proportion (%)	9.3	28.6	33.3	20.6	8.26	100
Area km^2^	3,220	9,964	11,592	7,176	2,876	34,828
population (thousands of people)	142.2	226.4	278.3	146.2	47.6	840.6

*Hyalys* antivenom, *Naja* antivenom, *Bungarus multicnctus* antivenom, *Agkistrodon Acutus* antivenom equipped hospital, and equipped with antivenom for all species of snakes in the country.

#### Accessibility Results of 21 Hospitals Equipped With *Agkistrodon Hyalys* Snake Antivenom in Hainan Province

**FIGURE 1:**
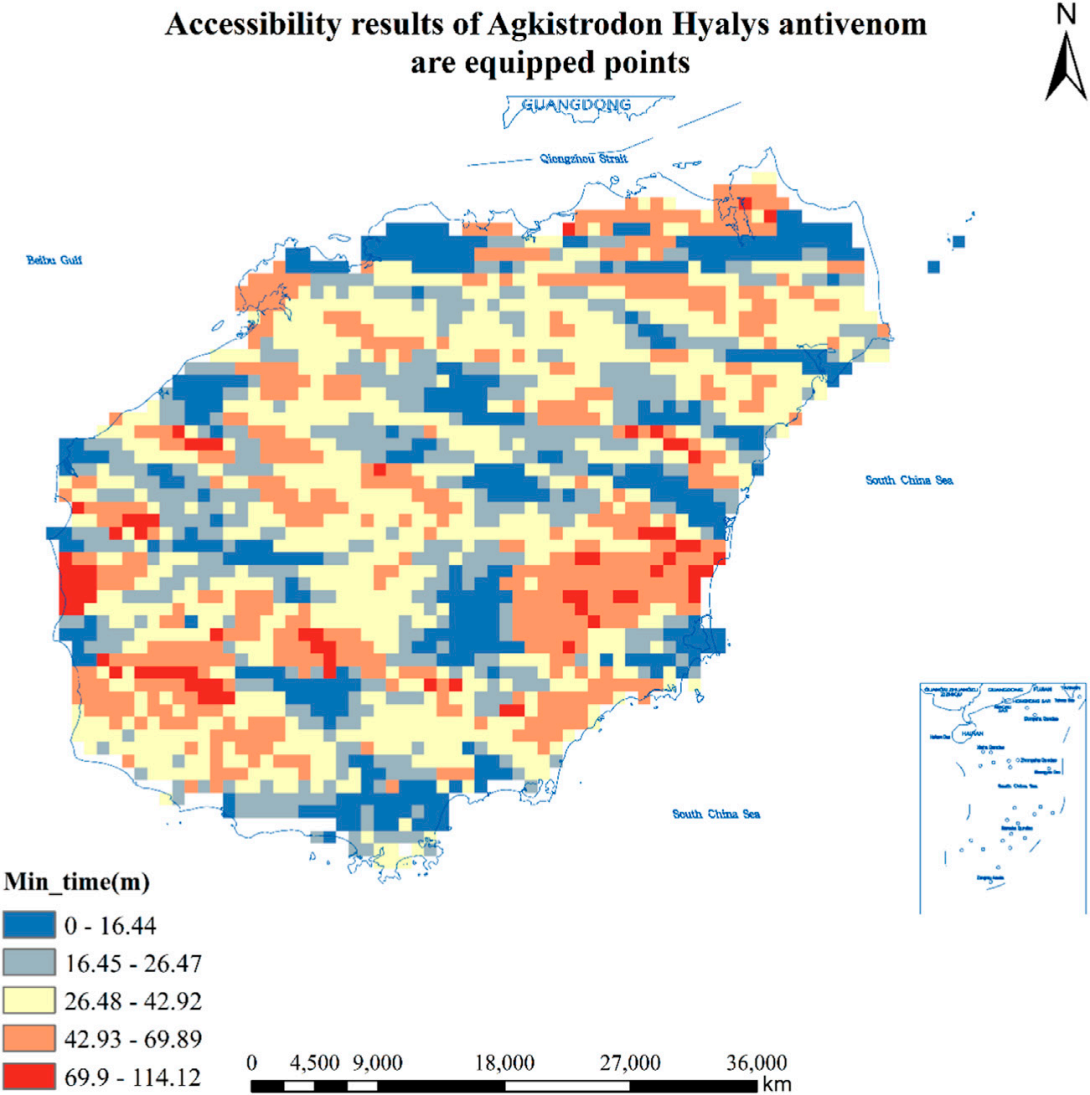
Accessibility results of 21 hospitals equipped with *Agkistrodon Hyalys* antivenom in Hainan Province (Haikou, China, 2024). Approval number: Joan S (2023) No. 254.

As can be seen from Table 1, the number of population centers of mass to locate hospitals for *Agkistrodon Hyalys* snake antivenom was 8,708, and the total number of population centers of mass that could reach the hospitals in less than 30 min was 3,855, which accounted for 44.3% of the population, and covered an area of about 15,420 square kilometers, or about 3,452,500 people; The total number of population centers of mass greater than 30 min and less than or equal to 60 min or less is 4,159, or 47.8%, covering an area of about 16,636 square kilometers and about 4,098,700 people; The total number of population centers of mass greater than 60 min and less than or equal to 90 min or less is 645, or 7.4%, covering an area of about 2,580 square kilometers and about 811,800 people; The total number of population centers of mass greater than 90 min and less than or equal to 120 min or less is 49, or 0.6%, covering an area of about 196 square kilometers and about 44,400 people. See Figure 1 for details.

#### Accessibility Results of 14 Hospitals Equipped With *Naja* Antivenin in Hainan Province

**FIGURE 2:**
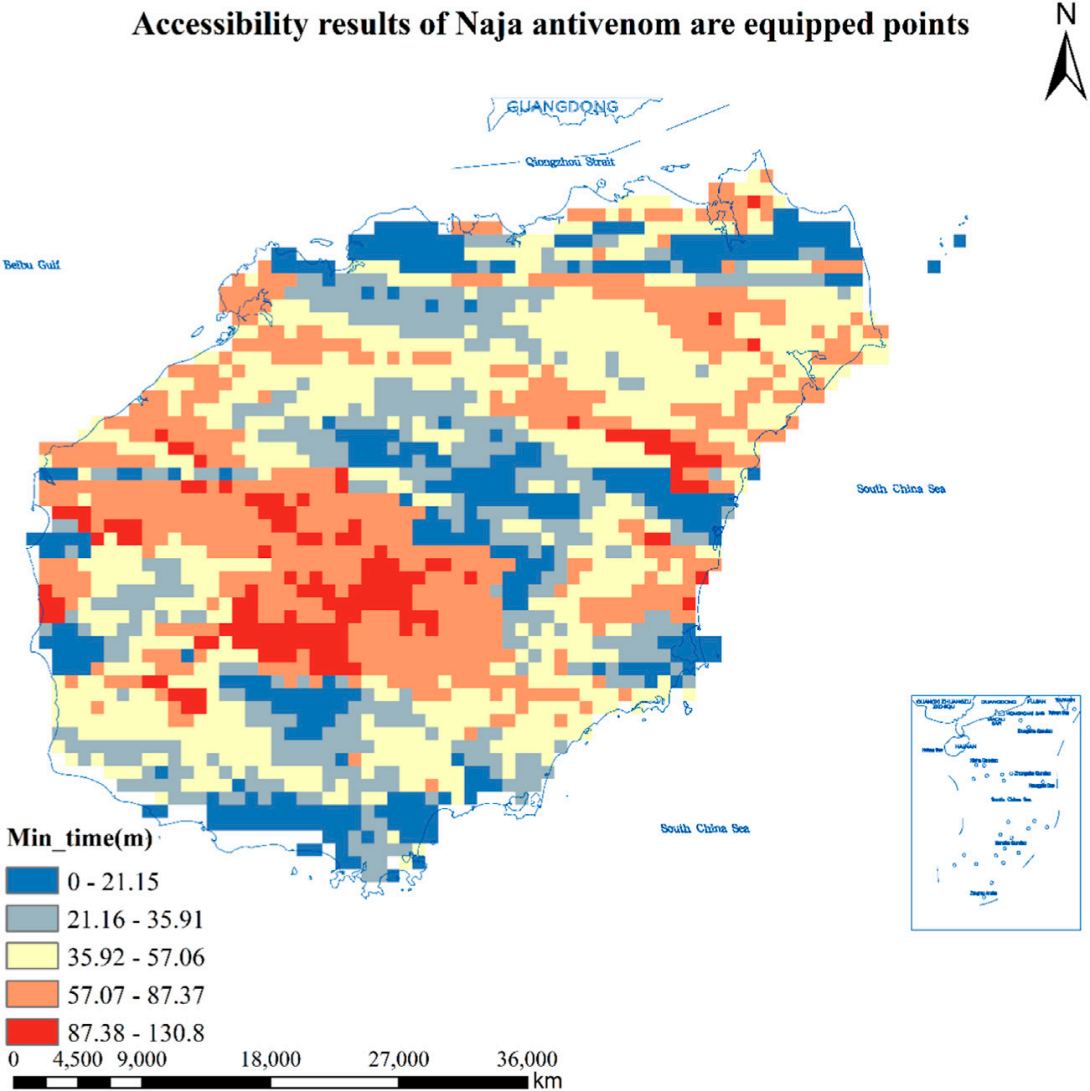
Accessibility results of 14 hospitals equipped with Naja antivenom in Hainan Province (Haikou, China, 2024). Approval number: Joan S (2023) No. 254.

As can be seen from Table 1, the number of population centers of mass that found hospitals equipped with *Naja* antivenom was 8,707, and the total number of population centers of mass that could reach the hospitals in less than 3 0 min was 2,118, or 24.3%, covering an area of about 8,472 square kilometers, or about 2,318,400 people; There are a total of 3,971 population centers of mass greater than 30 min and less than or equal to 60 min or less, accounting for 45.6% of the total, covering an area of about 15,884 square kilometers and about 3,775,500 people; There are a total of 2,153 population centers of mass greater than 60 min and less than or equal to 90 min or less, accounting for 24.7% of the total, covering an area of about 8,612 square kilometers and about 2,089,900 people; The total number of population centers of mass greater than 90 min and less than or equal to 120 min or less is 444, or 5.1%, covering an area of about 1,776 square kilometers and about 215,600 people; The total number of population centers of mass greater than 120 min is 21, or 0.2%, covering an area of about 54 square kilometers and about 0.7 million people. See Figure 2 for details.

#### Accessibility Results of 11 Hospitals in Hainan Province Equipped With Antivenom Against *Bungarus multicnctus* Antivenom

**FIGURE 3:**
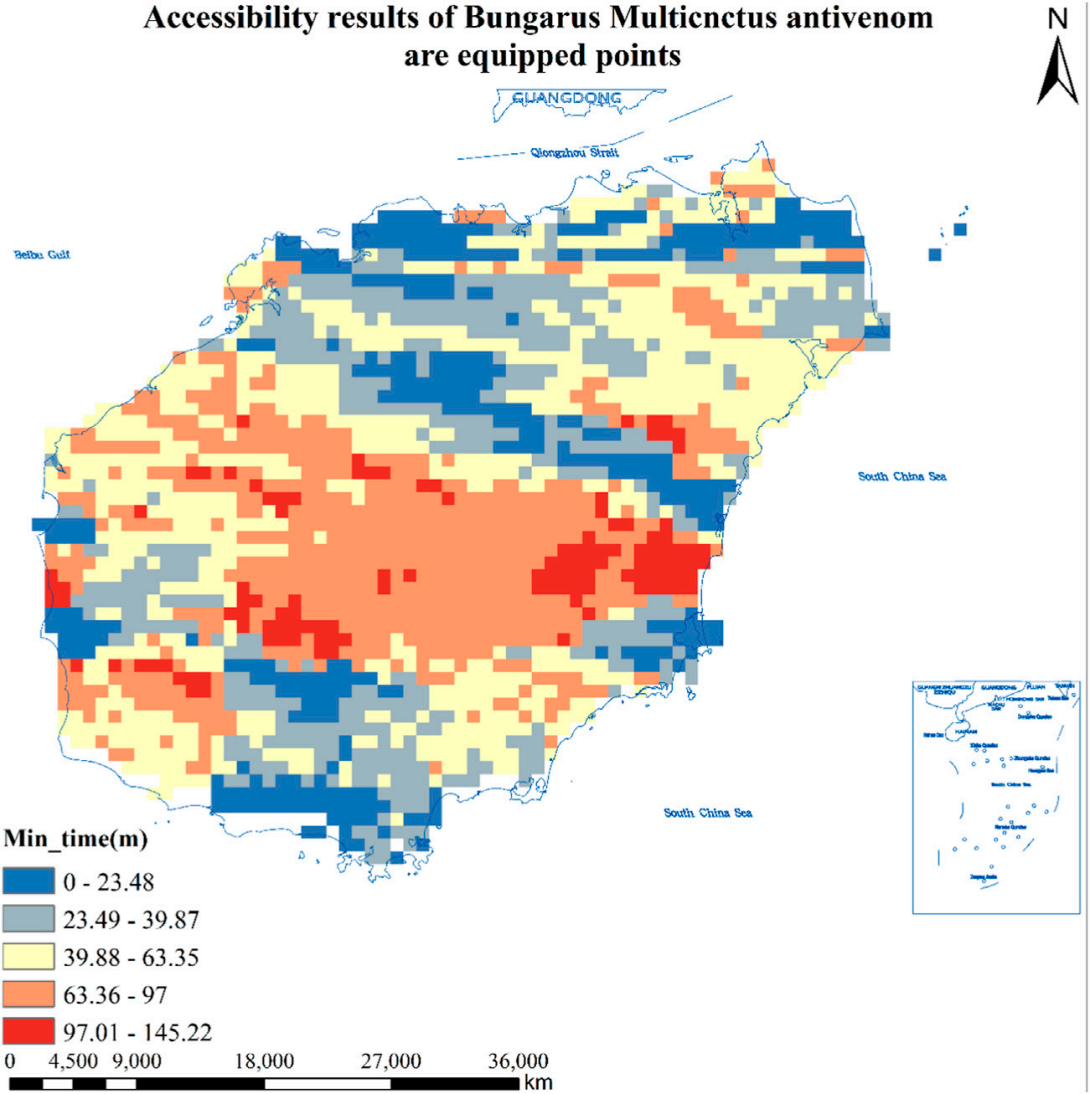
Accessibility results of 14 hospitals equipped with *Bungarus multicnctus* antivenom in Hainan Province (Haikou, China, 2024). Approval number: Joan S (2023) No. 254.

As can be seen from Table 1, the number of population centers of mass that found hospitals equipped with *Bungarus multicnctus* antivenom was 8,707, and a total of 1,799 population centers of mass that could reach the hospitals in less than 30 min, accounting for 20.7% of the total, covering an area of about 7,196 square kilometers and about 2,177,600 people; The total number of population centers of mass greater than 30 min and less than or equal to 60 min or less is 3,683, or 42.3%, covering an area of about 14,732 square kilometers and about 3,157,600 people; There are a total of 2,510 population centers of mass greater than 60 min and less than or equal to 90 min or less, accounting for 28.8% of the total, covering an area of about 10,040 square kilometers and about 2,516,900 people; There are 654 population centers of mass greater than 90 min and less than or equal to 120 min or less, accounting for 7.5% of the total, covering an area of about 2,616 square kilometers and about 516,700 people; The total number of population center of mass points greater than 120 min is 61, or 0.7%, covering an area of about 244 square kilometers and about 37,300 people. See Figure 3 for details.

#### Accessibility Results of 11 Hospitals Equipped With *Agkistrodon Acutus* Antivenom in Hainan Province

**FIGURE 4:**
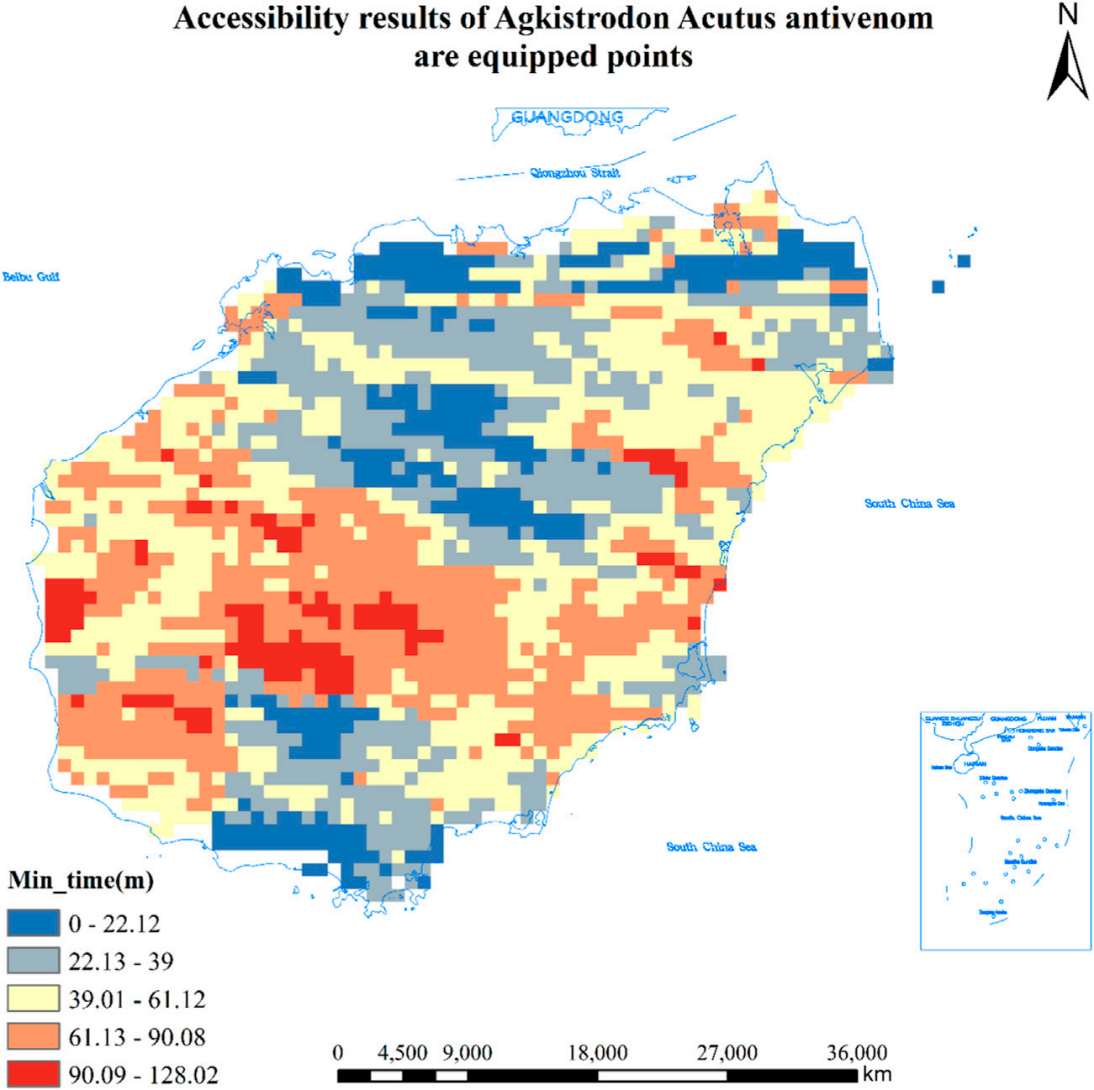
Accessibility results of 14 hospitals equipped with *Agkistrodon Acutus* antivenom in Hainan (Haikou, China, 2024). Approval number: Joan S (2023) No. 254.

As can be seen from Table 1, the number of population centers of mass to locate hospitals equipped with *Agkistrodon Acutus* antivenom was 8,707, and a total of 1,695 population centers of mass that could reach the hospitals in less than 3 0 min, accounting for 19.5% of the total, covering an area of about 6,780 square kilometers and about 2,240,900 people; The total number of population centers of mass greater than 30 min and less than or equal to 60 min or less is 4,045, or 46.5%, covering an area of about 16,180 square kilometers and about 3,498,900 people; There are a total of 2,521 population centers of mass greater than 60 min and less than or equal to 90 min or less, accounting for 29.0% of the total, covering an area of about 10,084 square kilometers and about 2,449,900 people; The total number of population centers of mass greater than 90 min and less than or equal to 120 min or less is 415, or 4.8%, covering an area of about 1,660 square kilometers and about 181,300 people; The total number of population centers of mass greater than 120 min is 31, or 0.4%, covering an area of about 124 square kilometers and about 35,200 people. See Figure 4 for details.

#### Accessibility Results of Five Hospitals in Hainan Province Equipped With Antivenom for All Species of Snakes

**FIGURE 5:**
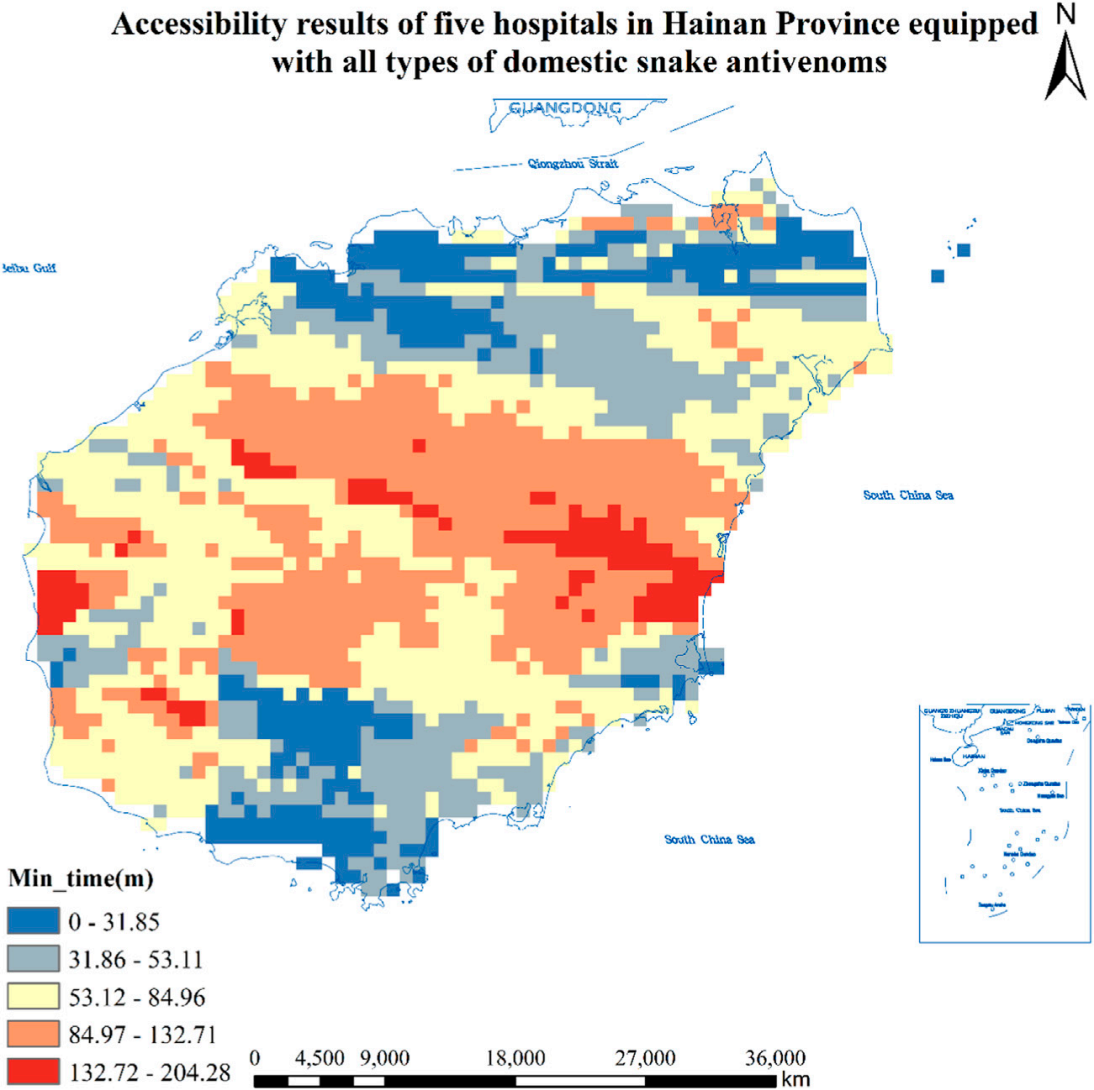
Accessibility results of five hospitals in Hainan Province equipped with all types of domestic snake antivenoms (Haikou, China, 2024). Approval number: Joan S (2023) No. 254.

As can be seen from Table 1, the number of population centers of mass where hospitals equipped with the antivenom for all species of snakes were found was 8,707, and the total number of population center of gravity where hospitals could be reached in less than 30 min was 805, or 9.3%, covering an area of about 3,220 square kilometers, or about 1,421,700 people; The total number of population centers of mass greater than 30 min and less than or equal to 60 min or less is 2,491, or 28.6%, covering an area of about 9,964 square kilometers and about 2,263,500 people; The total number of population centers of mass greater than 60 min and less than or equal to 90 min or less is 2,898, accounting for 33.3% of the total, covering an area of about 11,592 square kilometers and about 2,782,500 people; The total number of population centers of mass greater than 90 min and less than or equal to 120 min or less is 1,794, or 20.6%, covering an area of about 7,176 square kilometers and about 1,462,000 people; The total number of population centers of mass greater than 120 min is 719, or 8.3%, covering an area of about 2,876 square kilometers and about 476,400 people. See Figure 5 for details.

### Results of Accessibility of Antivenom Equipped Hospitals With 1,000 Beds and 1,000 Health Technicians

The presence of several hospitals within the southernmost city of Sanya and the northernmost city of Haikou in the study area creates a distribution of high values of accessibility within their boundaries, whereas the small number of antivenom-equipped hospitals in the other regions has relatively low values of accessibility. See Supplementary File 4. The presence of several hospitals within the southernmost city of Sanya and the northernmost city of Haikou in the study area creates a distribution of high values of accessibility within their boundaries, whereas the small number of antivenom-equipped hospitals in the other regions has relatively low values of accessibility. See Supplementary File 5.

## Discussion

### Accessibility Analysis of Hospitals Equipped With Snake Antivenom in Hainan Province

Venomous snake bites are medical emergencies that can be life-threatening [21]. Venomous snake bites can lead to different symptoms such as respiratory paralysis [22], swelling of limbs [23], tissue necrosis, profuse bleeding and tissue destruction [24]; in severe cases, death can result [21]. The most common chronic condition resulting from poor treatment outcomes is musculoskeletal, with severe cases involving tissue necrosis [25]. Antivenom is currently the only effective antidote, and early injection of antivenom after bite can prevent complications and adverse outcomes [26–29]. Snakebites are rarely fatal if the correct antivenom is obtained in a timely manner, but in countries without strong health systems and antivenom stockpiles, deaths and disabilities occur all the time [30]. For proper treatment, the World Health Organization recommends a maximum time limit of 1 h to reach an antivenom-equipped health facility [31]. Therefore, the accessibility of population center of mass points to various types of hospitals providing antivenom in Hainan Province was analyzed to identify areas where antivenom resources could be improved.

At present, there are four kinds of snake antivenom in China, and these kinds of antivenom have been stocked in Hainan Province, among which the most used ones are *Agkistrodon hyalys* antivenin and Agkistrodon acutus antivenin. The distribution of accessibility of hospitals equipped with *Agkistrodon hyalys* antivenin, *Naja* antivenin, *Bungarus multicnctus* antivenin, *Agkistrodon Acutus* antivenin, and all species of snake venom in the study area is uneven, generally weak in the central-southern region and better in other regions. The northern and southern regions have a high degree of accessibility with many facility sites. According to this study, it took more than 2 h for some residents of Hainan Province to reach hospitals equipped with snake antivenom, and previous scholars have found that delays in care for poisonings of more than 6 h after a venomous snake bite were associated with an increased risk of severe systemic venom stings, while arriving at a healthcare facility after 3 h was associated with an increase in the number of moderate and severe events [12]. This suggests that to reduce the severity of poisoning caused by venomous snake bites, it is necessary to reach a medical facility in time for effective treatment. Yet globally, nearly 2.7 million people are hospitalized each year for snakebite injuries, of which some 81,000 to 138,000 die, and some 400,000 are forced to have their limbs amputated or become permanently disabled [24]. There is a global shortage of antivenom, especially in developing countries, where poor healthcare systems and a shortage of pharmaceutical resources prevent many people from accessing safe and effective treatment. Inadequate availability of antivenom in parts of sub-Saharan Africa and Asia is an important factor in snakebite-related morbidity and mortality, and there is a need to improve access to antivenom [8], as is the case in parts of Hainan Province.

There are two major delays in the administration of antivenom that are currently understood. The first is any delay between the bite and arrival at a hospital where antivenom can be administered. The second is any delay between systemic envenomation (arrival of venom in the body’s circulation) and clinical recognition of systemic envenomation (signs or symptoms) or abnormal blood tests. Encouraging early attendance at primary healthcare centers is key to improving snakebite outcomes globally [2]. Hainan Province compares favorably with the Brazilian Amazon, where it can take more than 2 h for farmers and indigenous people to obtain life-saving antivenom immunizations [32]. In Costa Rica, where 7% of the population living in areas at high risk of snakebite takes more than 2 h to reach a hospital or clinic, the same imbalance in resource allocation exists. On the south Pacific coast around Golfo Dulce, around the Talamanca highlands in the southeast, and along the northern border, there are populations in high risk of snakebites and with long transportation times to antivenom treatment [33]. Of the children admitted to Limon Hospital on the Caribbean coast in 1985–1995, 50 per cent were treated within 3 h, compared with an average of 6.8 h, which suggests that some snakebite victims in the region have very long transportation times and high difficulty in accessing medical resources [33].

In a study in Ethiopia, only 5.2% of the antivenoms were available in 4 states [34]. In a prospective cohort study in Sri Lanka, the median time to antivenom treatment for all patients was 3.8 h. Although the majority of snakebite patients in the cohort presented to the hospital within 1 h of the bite, untimely diagnosis still resulted in a delay of approximately 2 h in the administration of the first dose of antivenom [35].

Poor accessibility of antivenom is an important factor in snakebite induced complications and mortality [8, 36], and snakebite is rarely fatal if the correct antivenom is obtained in time [30]. However, the poor supply of antivenom still exists in some areas of Hainan Province. Attention should be paid to optimizing the distribution of antivenom, which includes improving the production and distribution of antivenom, ensuring accessibility, strengthening healthcare infrastructure, and raising awareness of the importance of antivenom in the treatment of snakebite.

### Resource Accessibility Analysis of Snake Antivenom-Equipped Hospitals in Hainan Province

Sanya and Haikou cities in Hainan Province have a number of hospitals that provide antivenom within their boundaries, forming a region of high values of accessibility, while other regions have a small number of antivenom-equipped hospitals with comparatively low accessibility. The reason for this phenomenon is that the Gaussian two-step moving search method considers both supply and demand [37], and due to the fact that there are more hospitals equipped with antivenom within the jurisdiction of Haikou City and Sanya City, the supply is large, and it is possible to obtain the supply service of hospitals equipped with antivenom more conveniently, and the hospitals are of higher grades, with a large number of medical staff, and a large supply, and it is possible to obtain the supply service of hospitals equipped with antivenom more conveniently. This results in an accessibility high value distribution area. The other regions have fewer facilities and poorer hospital development than Haikou and Sanya, with less supply and greater demand compared to the other regions. In addition, according to data from Hainan’s tourism website, snakes are most abundant in the central region, which is surrounded on all sides by mountains, adequate rainfall, and scrub forests, which are suitable for the survival of snakes. This area is mainly covered with large areas of tropical rainforests, cash crops such as rubber and betel nut, and residents have relatively high exposure to snakes during outdoor work. Whereas the resources of antivenom in this area are limited, lack of antivenom treatment increases the risk of death in snakebite patients [38]. In this study, the combination of commuting distance, amounts of medical facilities, medical staff and demographic data can evaluate the accessibility of medical resources more objectively. Therefore, in order to improve the current *status quo* of antivenom resource allocation, resources could be targeted to vulnerable areas based on the results of the accessibility analysis.

The population size represented by the World Pop raster data in this study is corrected by street population data from the seventh population census of Hainan Province, 517 which has some limitations in accuracy. Precise demographic data should be captured within the subdivision to enhance the accuracy of the algorithm results. Factors such as traffic volume were not considered in the time cost analysis, and full consideration should be given to the conditions that a car may encounter while traveling in order to accurately reach the results of the accessibility analysis.

### Conclusion

This study shows that the accessibility of hospitals equipped with antivenom in Hainan Province is unevenly distributed, with the capital cities of Haikou and Sanya having the highest accessibility, while the central region lacks antivenom resources and has a low value of accessibility, and the *status quo* needs to be improved.
