# Corrigendum: Spatial Accessibility Analysis of Snake Antivenom

**DOI:** 10.3389/ijph.2026.1609456

**Published:** 2026-01-22

**Authors:** Wenjie Hao, Lanfen He, Xingyue Song, Juntao Wang, Yanlan Hu, Yu Chen, Chuanzhu Lv, Shijiao Yan

**Affiliations:** 1 Hainan General Hospital, Haikou, China; 2 School of Public Health, Hainan Medical University, Haikou, Hainan Province, China; 3 Second Affiliated Hospital of Hainan Medical University, Haikou, China; 4 Sichuan Academy of Medical Sciences and Sichuan Provincial People’s Hospital, Chengdu, China

**Keywords:** antivenom, healthcare organizations, accessibility, snakebite, snakebite envenoming

There was an error in affiliation **1**. Instead of “***Hainan Ceneral Hospital, Haikou, China***”, it should be “***Hainan General Hospital, Haikou, China***”. The authors apologize for this error and state that this does not change the scientific conclusions of the article in any way. The first published incorrect version of the article has been updated.

