# Supplementary material for: Spatial Accessibility Analysis of Snake Antivenom
Source: Int J Public Health. 2025 Jan 3;69:1606903. doi: 10.3389/ijph.2024.1606903 (PMC11738613; doi:10.3389/ijph.2024.1606903)
Supplement: Supplementary file 1 [file DataSheet4.docx]

**Supplementary File 4**


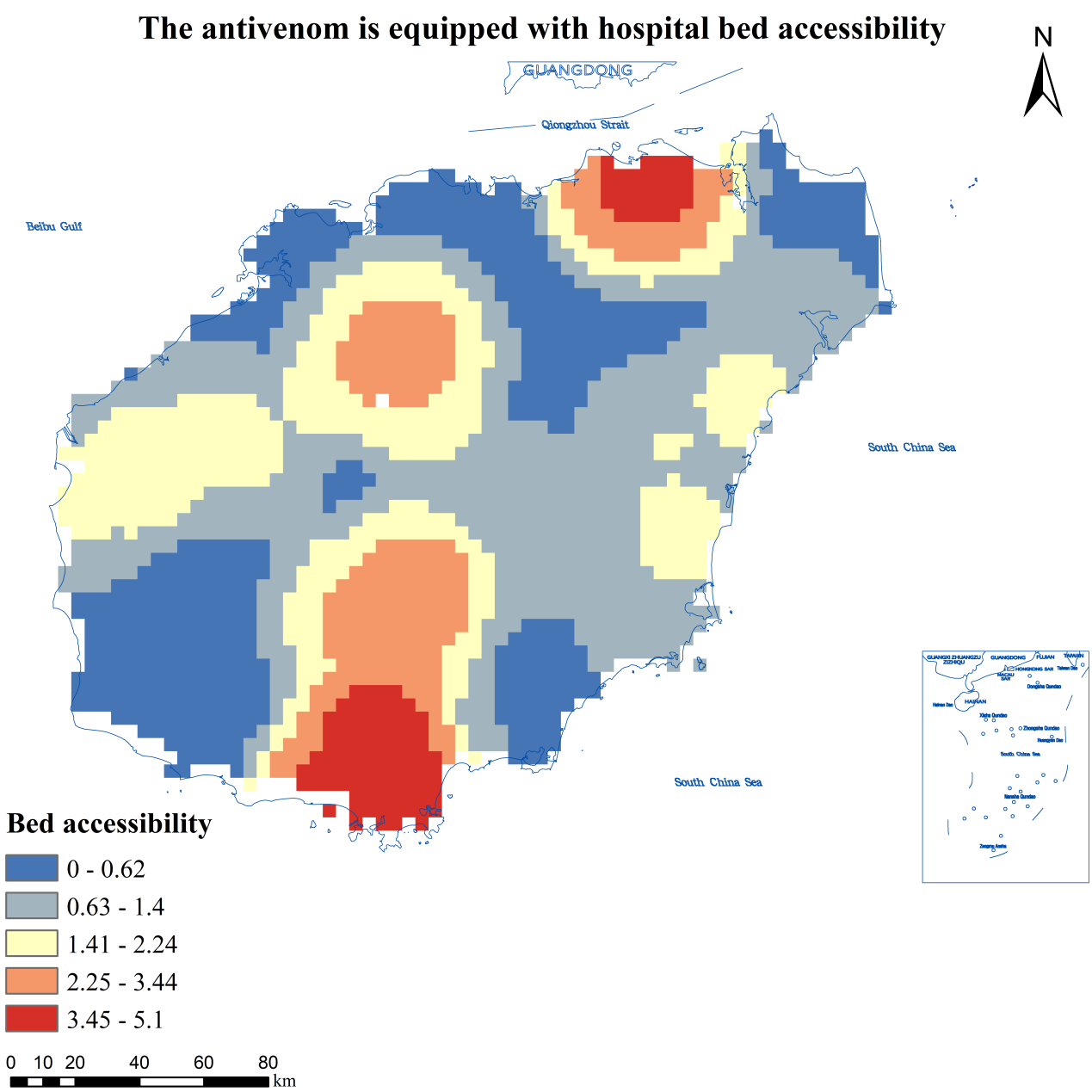


Figure Results of accessibility of 1,000 beds in 25 antivenom-equipped hospitals in Hainan Province, China（Haikou, China, 2024）

Approval number: Joan S (2023) No. 254
