# Supplementary material for: Spatial Accessibility Analysis of Snake Antivenom
Source: Int J Public Health. 2025 Jan 3;69:1606903. doi: 10.3389/ijph.2024.1606903 (PMC11738613; doi:10.3389/ijph.2024.1606903)
Supplement: Supplementary file 2 [file DataSheet3.docx]

**Supplementary File 3**

Table 1 Distribution of hospitals equipped with snake antivenom（Haikou, China, 2024）

| Region | Number of medical institutions equipped with antivenom | Proportion % |
| --- | --- | --- |
| Haikou City | 7 | 28 |
| Sanya City | 5 | 20 |
| Dan Zhou City | 1 | 4 |
| Baisha Li Autonomous County | 1 | 4 |
| Bao Ting Li and Miao Autonomous County | 1 | 4 |
| Changjiang Li Autonomous County | 2 | 8 |
| Dong fang City | 1 | 4 |
| Qiong Hai City | 1 | 4 |
| Qiong Zhong Li and Miao autonomous county | 1 | 4 |
| Tun Chang County | 1 | 4 |
| Wanning City | 1 | 4 |
| Wenchang City | 1 | 4 |
| Wu Zhi Shan City | 2 | 8 |
| Total | 25 | 100 |
