# Supplementary material for: Spatial Accessibility Analysis of Snake Antivenom
Source: Int J Public Health. 2025 Jan 3;69:1606903. doi: 10.3389/ijph.2024.1606903 (PMC11738613; doi:10.3389/ijph.2024.1606903)
Supplement: Supplementary file 4 [file DataSheet2.docx]

**Supplementary File 2**


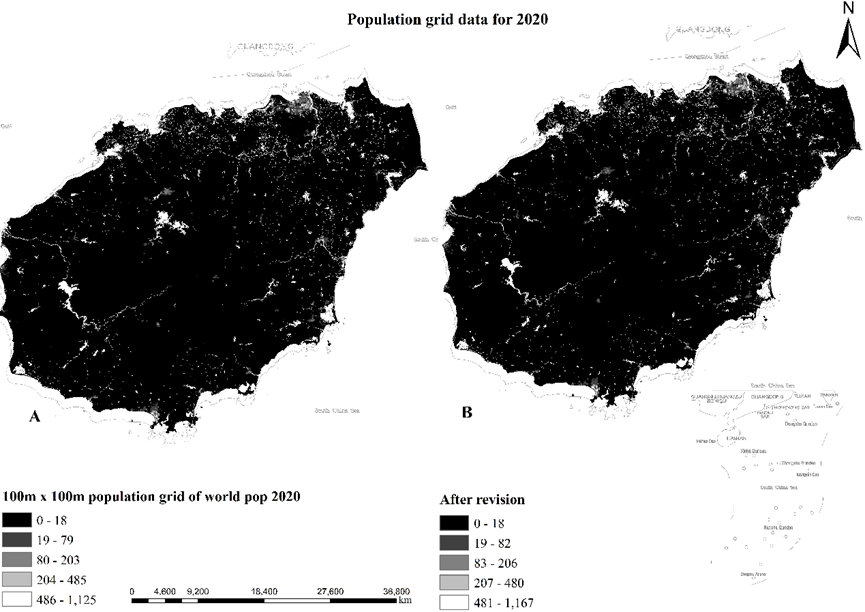


Population grid of Hainan Province in 2020（Haikou, China, 2024）

Approval number: Joan S (2023) No. 254

Note: Figure A is the 2020 World Pop population raster; Figure B is the population raster corrected for 7th Census data.
