# Supplementary figures and images for: Spatial Accessibility Analysis of Snake Antivenom
Source: Int J Public Health. 2025 Jan 3;69:1606903. doi: 10.3389/ijph.2024.1606903 (PMC11738613; doi:10.3389/ijph.2024.1606903)

**Supplementary File 1**


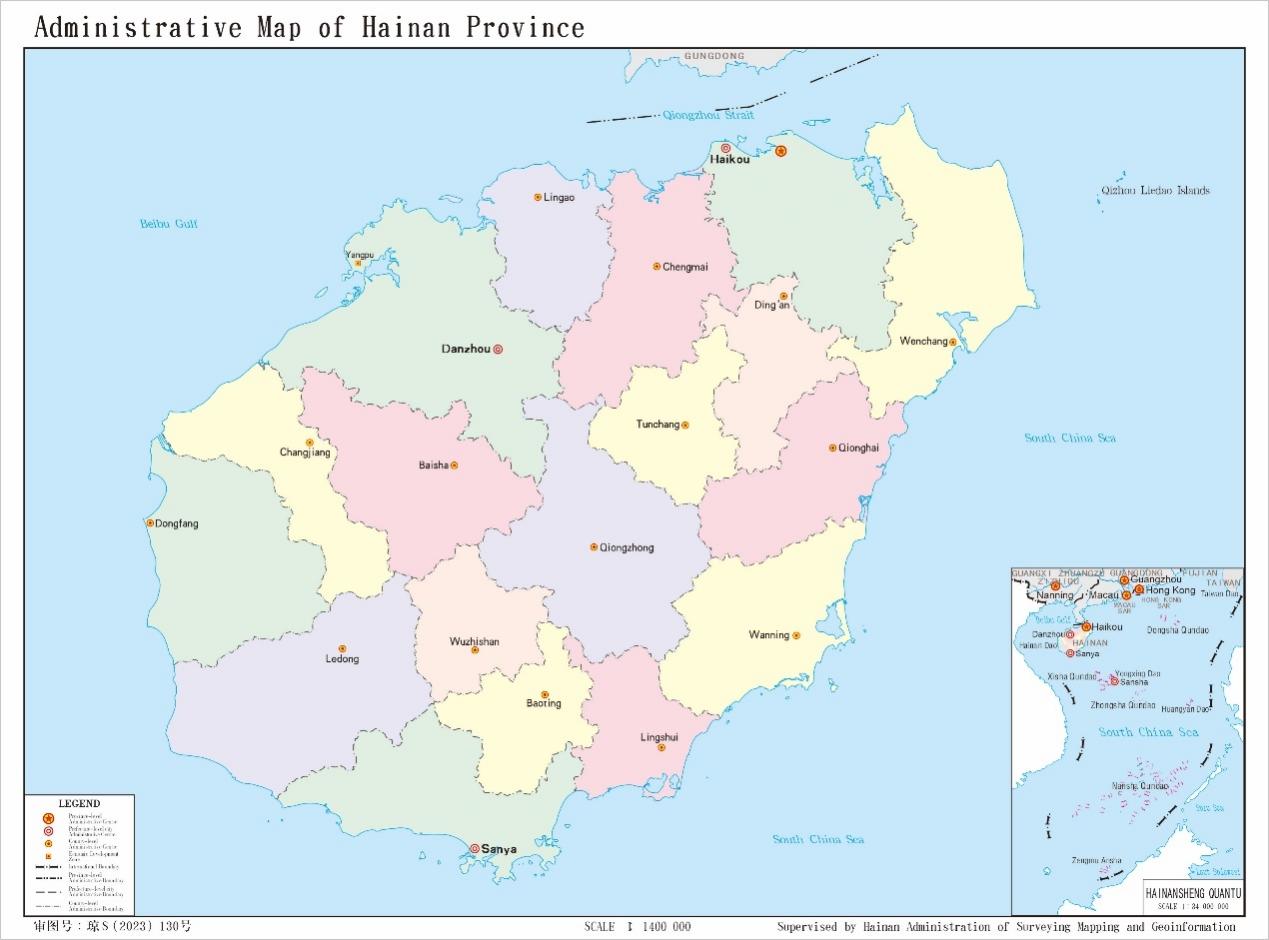


Map of Hainan Province Administrative Areas（Haikou, China, 2024）

Supplement: Supplementary file 5 [file DataSheet1.docx]
